# Decreased Sarcopenia in Aged Females with Young Ovary Transplants was Preserved in Mice that Received Germ Cell-Depleted Young Ovaries

**DOI:** 10.3390/jcm8010040

**Published:** 2019-01-03

**Authors:** Tracy L. Habermehl, Jeffrey B. Mason

**Affiliations:** Department of Animal, Dairy and Veterinary Sciences, Center for Integrated BioSystems, School of Veterinary Medicine, Utah State University, 4700 Old Main Hill, Logan, UT 84322, USA; tracy.habermehl@aggiemail.usu.edu

**Keywords:** germ cell-depleted (GD), germ cell-containing (GC), ovarian somatic cells, menopause, sarcopenia

## Abstract

Previously, transplantation of young, cycling, ovaries increased life and health span in post-reproductive female mice. The current study addressed the influence of ovarian germ cells in the improvement in health by performing transplantations of young, germ cell-depleted ovaries. The purpose of this study is to further the understanding of reproductive influences on aging health. Control mice were grouped by age. Treatment mice were age-matched and received either germ cell depleted ovaries or germ cell containing ovaries at 400 days of age. All groups underwent health span assays until sacrifice (treatment and age-matched control groups were between 680 and 700 days). Body composition results displayed an improvement of body composition in both treatment groups, compared to the controls, but no significant difference between the germ cell-depleted or germ cell-containing groups. Grip test results showed no improvement in musculoskeletal endurance and no change to mild loss of grip strength with both transplant groups compared to control groups. The research presented here suggests that reproductive status has a positive influence in post-reproductive health. A portion of this influence may be germ cell independent.

## 1. Introduction

The preservation of health during aging is dependent upon several factors. In women, menopause, or reproductive decline, enormously increases the risk for disease including several musculoskeletal conditions. Sarcopenia, or the decline in skeletal muscle mass, is a common condition associated with aging. The weakness in skeletal muscle can make daily activities tiresome and difficult. Reproductive status can have a positive influence on health. In primitive organisms, the depletion of germ cells extends longevity [1]. In more recent studies, the transplantation of young ovaries into post-reproductive mice extend longevity and improves health, including preservation of lean muscle mass, decreased cardiomyopathy and decreased osteoarthritis [2,3,4]. Combining young ovary transplantations and germ cell depletion (as seen previously in primitive organisms), we transplanted young, germ cell-depleted ovaries into post-reproductive mice. Longevity in these mice was extended further than the longevity extension in mice that received germ cell-containing transplants [5]. Little is known as to what influence the lack of ovarian germ cells and presence of young, ovarian somatic cells has on post-reproductive health. In the current manuscript, we investigated the influence of the young, germ cell-depleted ovarian transplants on post-reproductive health in mice. We hypothesized that the lack of ovarian germ cells stimulates the ovarian somatic cells to support the organismal health rather than the germ cells, therefore restoring skeletal muscle.

## 2. Experimental Section

### 2.1. Animals

Female mice of the CBA/J strain, a common laboratory mouse used in aging studies, were used in this study due to their interesting process of ovarian follicle loss earlier in their lifespan, around 300 days of age [6,7,8]. The loss of ovarian follicles is comparable to the reproductive physiological process at menopause in humans. Therefore, the CBA/J strain of mice was an excellent candidate for a model organism in the study of age and menopause associated diseases and conditions.

Female CBA/J mice of 75 days of age, 200 days of age, 450 days of age, and 500 days of age were obtained from Jackson Laboratory (Bar Harbor, ME, USA). The mice were housed individually in ventilated cages (Green Line IVC Sealsafe Plus, Tecniplast, West Chester, PA, USA). The laboratory environment consisted of fresh filtered air (15 changes/h), temperature of 21 ± 2 °C, humidity of 50 ± 20%, and a light-dark cycle (12:12 h) in a specific pathogen free colony. Each cage contained corncob bedding (7097 Corncob, Harlan Teklad, Bartonville, IL, USA), deionized water, laboratory rodent diet *ad libitum* (2018 Teklad Global 18% Protein Rodent Diet, Harlan Teklad, Bartonville, IL, USA), and added enrichments.

Mice were maintained in an American Association for Accreditation of Laboratory Animal Care (AAALAC)-approved facility in accordance with the National Institutes of Health animal-use guidelines. Animal care and use protocols were developed under the National Research Council guidelines found in the Guide for the Care and Use of Laboratory Animals. This project was approved by the Utah State University Institutional Animal Care and Use Committee (IACUC-2277).

Anesthetics were used during surgery for both the donor and recipient mice. Analgesia was provided to the recipient mice for 48 h post operation or longer if necessary. Euthanasia of donor mice occurred via cervical dislocation. A thoracotomy was performed immediately after cervical dislocation and followed by rapid exsanguination via cardiocentesis. Mice with acute weight loss were treated with moistened food and subcutaneous fluids. Animals with acute urine staining or rectal/vaginal prolapse were manually cleaned and treated with Desitin^®^. Mice were monitored at least twice daily, weights were recorded at least monthly and more frequently if concerns appeared. Moribund, aged mice whom exerted overt clinical signs (catatonia) were euthanized. Criteria for euthanasia specifically for aged mice was determined in coordination with the attending veterinarian and included, but not limited to, mice found in poor condition with or without crusting around the perineum and diarrhea, urine staining, persistent vaginal prolapse, chronic vulva/rectal swelling, kyphosis, respiratory distress, anorexia, poor coat condition with lack of grooming, moribund mentation, hind-limb paresis, wounds not healing, neoplastic growths, and unusual weight loss or gain. Aged female CBA/J mice have an average weight loss from peak weight to death of 12 percent per month [9]. An increased rate of weight loss, but not total weight loss, was the most critical factor for determining a moribund state. Unexpected deaths were uncommon but included neoplastic growths, decubitus ulcers, and uncontrolled cataleptic seizures. Mice who received GC transplants, or germ cell-containing young ovaries, in previous studies lived an average of 770 days. The Mice who received GD transplants lived an average of 898 days [3]. The mice in this study were euthanized at ages 200, 300, 700, and 900 days for the control groups and 700 days of age for the treatment groups (10 days).

### 2.2. Experimental Design

**Figure 1:**
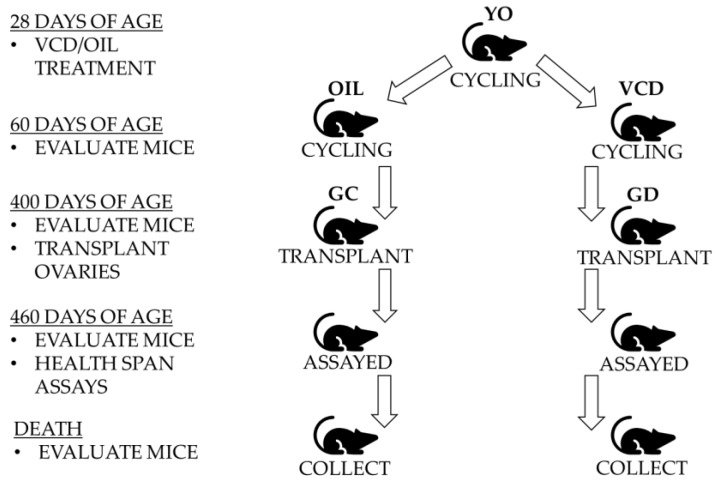
Experimental Design. Twenty-eight-day mice with young ovaries (YO) were treated with 4-vinylcyclohexene diepoxide (VCD) or Oil for 15 days. At 60 days of age, germ cell-depleted (GD) ovaries and germ cell-containing (GC) ovaries were removed and transplanted into 400-day mice. After a minimum of one month to recover, all mice were evaluated and assessed via health span assays starting at 460 days of age. Mice were collected when the treatment groups were 700 ± 10 days for further health span analysis and evaluation.

Four-hundred-day old female CBA/J mice were randomly selected as control, germ cell containing transplant recipients, and germ cell depleted transplant recipients (Figure 1). The different groups and their ages at the time of each assay and euthanasia are presented in Table 1.

#### 2.2.1. Control Groups

The control groups of mice consisted of the following individuals that were separate groups of mice, not repeated measures.

- One-hundred-day old mice that were reproductively cycling (*n* = 19).
- Two-hundred-day old mice that were both cyclic and acyclic during the study (*n* = 10).
- Four-hundred-day old mice that were acyclic (*n* = 5).
- Six-hundred day old mice that were acyclic and from a previous study (*n* = 3).

#### 2.2.2. Experimental Groups

The ovarian transplant recipient mice consisted of the following.

- Four-hundred-day old mice, that were acyclic at the time of surgery, received 60-old ovaries from donor mice that were cycling (*n* = 9).
- Four-hundred-day old mice, that were acyclic at the time of surgery, received 60-day-old germ cell-depleted (GD) ovaries from donor mice that were cycling (*n* = 11).

### 2.3. Age at Ovarian Manipulation

Donor mice of 28 days of age were randomly chosen to receive placebo injections of sesame oil or treatment with 4-vinylcyclohexene diepoxide (VCD) to deplete the germ cells from the young ovaries (*n* = 20). The germ cell-depleted donor mice received intraperitoneal injections of 160 mg/kg VCD daily for 15 days. A 15-day VCD-dosing protocol was previously established to stop reproductive cycling and deplete ovarian primordial and primary follicles [10].

Rodents do not undergo menopause, but instead they have and estropause-like decrease in reproductive function. Reproductive decline in CBA/J mice typically begins at 240 days of age and most, if not all, are reproductively incompetent by 330 days of age [11]. Female CBA/J mice become reproductively active between 45 and 60 days of age. Manipulation (VCD treatment) was initiated at 28 days of age to avoid dramatic up-regulation of the reproductive system at the onset of puberty and to eliminate alternative influences the ovaries might have in addition to the direct hormonal effects.

### 2.4. Ovarian Transplants

Female CBA/J mice become reproductively active between 45 and 60 days of age. Donor mice were anesthetized, and ovaries were extracted when they reached 60 days of age and were actively cycling. Cycles were analyzed using microscopic analysis of vaginal lavage. After ovarian removal, the donor mice were euthanized using the protocols mentioned previously.

Recipient mice were anesthetized and underwent ovarian transplant surgery using the donor ovaries as described in [10,11,12]. Briefly, a vertical incision was made in the lateral aspect of the abdomen between the coxa and the ribs. The ovarian fat pad was identified and removed into a field of view outside of the body cavity. A small opening was made in the ovarian bursa and the ovary was extracted. The donor ovary was then placed into the emptied bursa. The bursa and abdominal cavity were sutured and the skin stapled closed. This was performed on both the left and right ovaries. All recipient mice were allowed one month of recovery before assays were performed.

### 2.5. Body Composition Assay

Mice were weighed at the same time daily for three consecutive days leading up to the magnetic resonance imaging (MRI). Whole body composition changes were assessed with an EchoMRI-700 Magnetic Resonance Imaging system (EchoMRI, Houston, TX, USA). The MRI system is housed in a dedicated area to minimize stress during the procedure. Prior to each run, the system was calibrated using a mouse standard provided by Echo Medical System. This MRI system uses the combination of the relaxation time of hydrogen protons and radio pulses to produce radio signals that create a contrast between the soft tissues in the body [13]. Due to the signals relying on several aspects of the pulses such as amplitude, duration and distribution, movement of the individual can alter the signals that are identified. The mouse holders are therefore used to contain the mouse in a position where there is minimal movement to get the most accurate readings on a conscious animal. Each mouse was then placed into the appropriately sized tube and then placed in the system for imaging (~60 s). The output data included adipose tissue mass, lean tissue mass, free water, and total body water. No anesthesia was needed for this procedure [2].

### 2.6. Musculoskeletal Endurance Assay

The overall muscular strength and endurance of each mouse was assessed using the Inverted-Cling Grip Test (ICGT) [14]. The apparatus used a 43 cm square wire mesh with 12 mm squares of 1 mm diameter wire surrounded by 4 cm deep wooden border. A padded surface was placed underneath the mesh for the mice to land on when they lost their grip. Each mouse was placed in the middle of the inverted screen, parallel to the floor. The screen was tilted up to a 90-degree angle allowing the mouse to grip onto the mesh. The screen was then tilted 90 degrees further making the screen parallel with the floor which positioned the mouse upside down where she was hanging suspended from the screen. The latency before the mouse lost her grip and fell to the padded surface was recorded for a maximum of one hour for three consecutive days. The assay was performed at the same time every day to eliminate any influence from circadian rhythms.

### 2.7. Hindlimb and Forelimb Musculoskeletal Strength

Hindlimb and forelimb grip strength was measured using a classical grip test with an Animal Grip Strength System (San Diego Instruments, San Diego, CA, USA) that consisted of dual mesh grids to measure front and rear limb strength in one run [15]. Each mouse was held by the base of their tail and placed on the apparatus where their forelimbs and hindlimbs were on separate, horizontal mesh grids. The mice were then pulled gently, yet fluidly, in a horizontal direction making sure the front paws did not touch the hindlimb grid. The grip strength system recorded the maximum force (grams-force) using a Chatillon (10-LBF) force gauge. Each mouse was tested five continuous time for three days.

### 2.8. Statistical Analysis

Statistical Analysis was performed with GraphPad Prism 7.04 (GraphPad Software, Inc., La Jolla, CA, USA). A D’Agostino-Person omnibus test was performed to determine normality. The data was analyzed with a two-factor ANOVA and a Tukey-Kramer post-hoc test was used to determine differences between groups. Student’s two-tailed *t*-test was performed on individual treatments assuming unequal distribution of variance. Any test result with a *p* Values less than 0.05 was considered significant. Each figure contains letters of significance above the data bars. The same lettered bars represent non-significant differences and different letters represent a statistically significant difference.

## 3. Results

All mice were separate groups with the same sample sizes throughout the experiment (minus 1–3 that were euthanized due to health conditions or passed randomly). These were not repeated measures.

### 3.1. Body Composition

**Figure 2:**
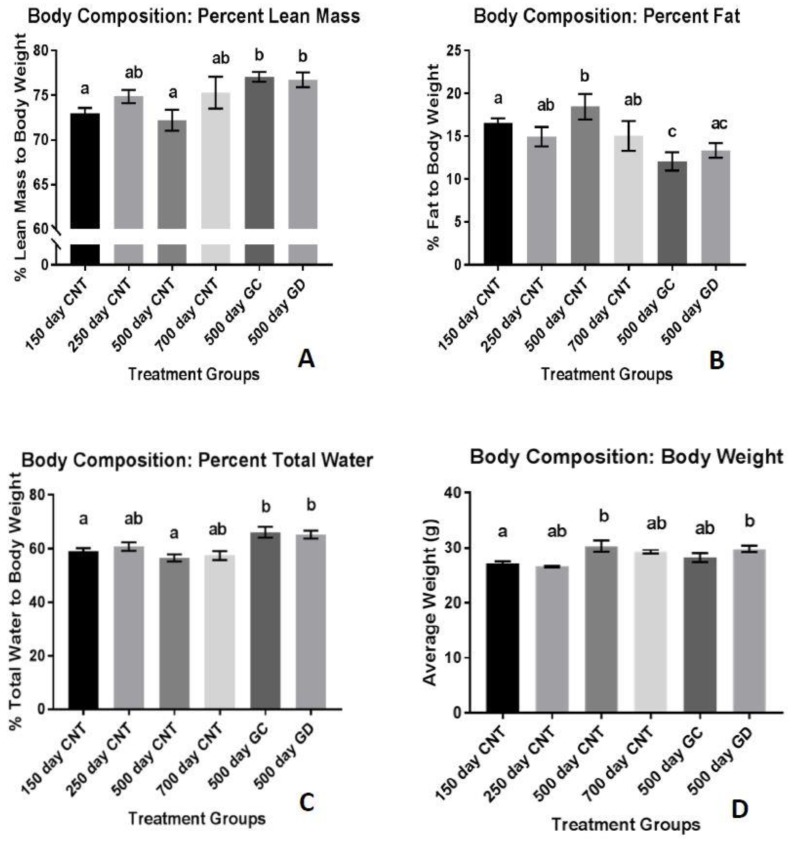
Body Composition: (**A**) Percent lean mass to body weight. There was a significant increase in lean mass with the treatment groups. (**B**) Percent fat mass to body weight. There was a significant decrease in fat mass with the two treatment groups. (**C**) Percent total body water to body weight. Both treatment groups had a significant increase compared to the youngest and age-matched control groups. (**D**) Average body weight. Body weight changed with age but not with treatment. Error bars are statistical standard errors. The CNT are control groups, GC are germ cell containing transplant recipients and GD are germ cell-depleted transplant recipients.

The percent fat mass, normalized to body weight, was significantly reduced in the GC and GD groups compared to the age-matched control group (*p* < 0.01 and *p* < 0.05 respectively) displayed in Figure 2A. There was no significant difference in the percent fat mass between the GC and GD groups. The percent of lean mass was significantly increased in the GC and GD transplant groups, compared to the age-matched control group (*p* < 0.05). There was also a significant increase in percent lean mass between the transplant groups and the 150 days old control group (*p* < 0.01 Figure 2B). There was no significant difference in lean mass between the two transplant groups. The total percent body water was consistently 60%–70% between the groups (Figure 2C). The treatment groups did have a significant increase in percent body water compared to the age-matched control group and the youngest group shown in Figure 2C (*p* < 0.01 and *p* < 0.05 respectively). Average body weight of the mice stayed between 25 and 30 g but both the GD and the age-matched control group weighed more than the youngest group. (Figure 2D, *p* < 0.05). The 700 days CNT is the oldest control group in Figure 2A and showed that the percent lean mass is increased compared to the age-matched control group (500 days CNT). When you compare this data to the other graphs in Figure 2, we suspect that the percent lean mass appears elevated due to the lower body weight and percent adipose tissue compared to the age-matched control group.

### 3.2. Musculoskeletal Endurance

**Figure 3:**
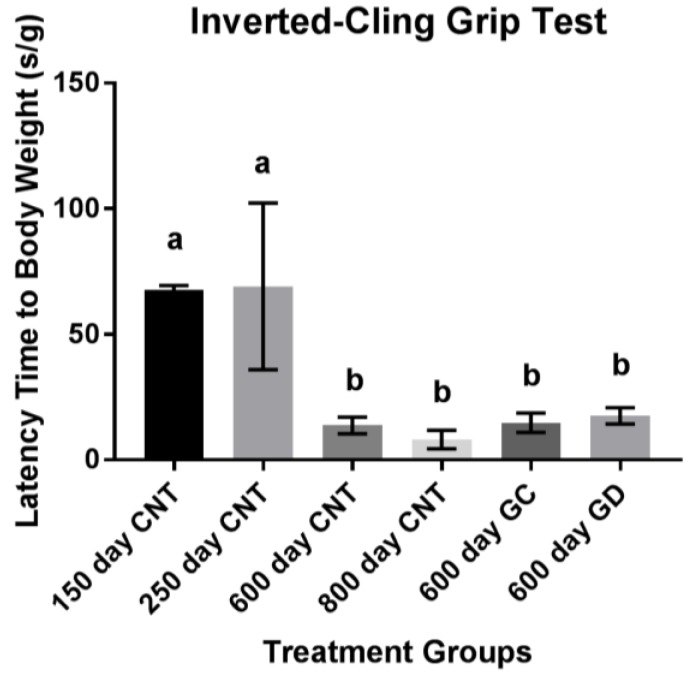
Musculoskeletal Endurance using Inverted-Cling Grip Test. No significant differences between the treatment groups, 600-day CNT, and 800-day CNT groups. The youngest two groups held on significantly higher than all other groups as expected (CNT represents the control groups of mice). Bars with the same letters above them indicate they are not significantly different from one another. Different letters indicate a statistically significant difference.

Musculoskeletal endurance, measured in latency of time to fall in seconds, declined with age. There was no statistical difference between the GC and GD transplants and no difference between the transplant groups and the age-matched control group and the old control group (Figure 3). The GD group did have a 3.7% increase in hang time compared to the age-matched control group and a 2.7% increase compared to the GC group. The GC group had a 1% increase in endurance compared to the age-matched control group. There were no trends noted (*p* > 0.1).

### 3.3. Musculoskeletal Strength

**Figure 4:**
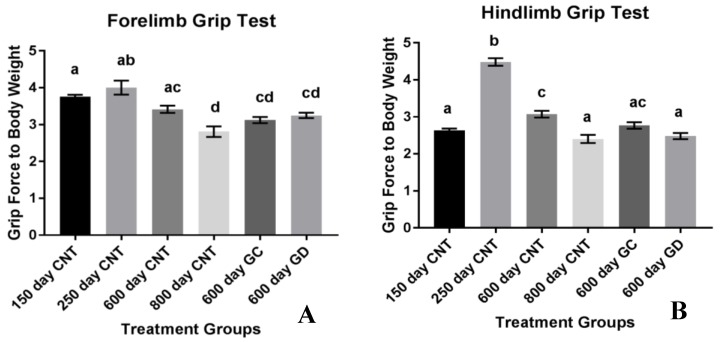
(**A**) Musculoskeletal Strength using the Classic Grip Test. Forelimb grip strength with standard error bars. There was a decline in forelimb grip strength with age but no significant difference with the treatment groups compared to the age-matched control group. (**B**) Hindlimb grip strength with standard error bars. There was also a decline in hindlimb strength with the treatment groups. There was a statistically significant decline with the GD group compared to the age-matched control group. Bars with the same letter(s) are not statistically different while bars with different letters are statistically different.

Musculoskeletal strength was also measured with grams of force exerted by the forelimbs and hindlimbs when pulled from the wire grid with respect to body weight. The forelimb strength of the mice declined with age. There was no difference between the forelimb strength of the transplant groups and the age-matched control groups. There was also no difference between the two treatment groups (Figure 4A). The musculoskeletal strength of the hindlimbs increased between the young control groups and then declined with age as shown in Figure 4B. There was a significant decline in hindlimb strength between GD and the age-matched control groups. There was no significant difference between GC and age-matched control group as well as between the transplant groups.

## 4. Discussion

Human aging normally leads to a decrease in muscle mass and an accumulation of adipose tissue mass. Decreased lean muscle mass has also been attributed to loss of ovarian function in post-menopausal women [16]. Based on previous studies involving worms [1], we hypothesized that the young ovarian somatic tissue is needed for the preservation and extension of health. In the current study, body weight slightly increased with age but stayed consistent between 25 and 30 g. Contradictory to other studies [9], the GC transplants did not display significantly reduced body weight. The GD transplants, which was the novel aspect of this study, were not significantly different in body weight, compared to the GC transplants. This suggests that ovarian germ cells may not play an exclusive role in the overall body weight control of female mice. Uniquely, lean mass and adipose tissue mass was significantly changed with the transplants. While body weight stayed consistent, lean mass significantly increased, even compared to the youngest group, while fat mass was significantly reduced in the transplant groups. Between the two treatment groups, neither fat mass or lean mass was statistically different suggesting that lean and fat mass may be less influenced by germ cell hormones and more influenced by somatic cell support. In human studies, lean mass increases have been noted with hormone replacement therapies (HRT) [17] while others have noted a decrease in lean muscle mass with HRT [18]. The contradictory studies suggest that HRT to treat sarcopenia may be variable. Our current research, suggesting that ovarian germ cells have little influence on body composition, provides novel evidence as to why HRT may be so unpredictable for the treatment of sarcopenia.

The germ cells and the cells most associated with the oocyte produce aromatase which converts the androgens into estrogens. The transplanted mice had an increase in percent lean mass with or without the germ cells suggesting that hormones may not assist in the treatment of sarcopenia as much as we originally thought. Menopause has been associated with an increase in fat mass in the body. The changes to the fat mass in the body may be associated with the hormone modifications occurring in post-menopausal women [19]. The treatment groups in this study showed a significant reduction in percent fat mass. With no difference between the GD and GC groups, the alterations in fat mass in these mice may be influenced by the germ cell independent mechanisms of restoring health, possibly more so than hormones. This provides evidence, with further research, that an ovarian somatic cell-based treatment, rather than HRT may have a more positive influence on body composition. One flaw to this current study is the assessment of hormone levels from the mice. Androgen and estrogen levels will be assessed in the future to confirm these ideas. Cycling status was check on a regular schedule by vaginal lavage cytology.

Percent total body water followed the trend of decreasing with age in the mice similar to post-menopausal women. Ovarian transplant recipients in the current study, as in other studies, increased their percent total body water, suggesting that young ovary transplantation restores the body to a physiologically younger state, even more so than the 150-day control group. There was little total body water difference between the GC and GD transplants. This may suggest that body composition is not necessarily dependent upon ovarian germ cells, but rather may be dependent upon the youthful somatic cells of the young ovaries.

Additionally, skeletal muscle mass and function decline with age [20]. Because of this decline, daily life activities can become difficult. Therefore, skeletal muscle has a large impact on quality of life and the assessment of muscle mass and function are necessary for an indication of health span. To assess the skeletal muscle function in this current study, two grip tests were performed. The inverted-cling grip test was chosen to assess the overall strength and endurance of the mice, similar to the pull-up test in humans [21]. In previous studies involving C57BL/6 mice, a black mouse used often in aging studies, total grip strength and endurance decreased with age. In our study with CBA/J mice, total grip strength and endurance also declined with age. There was a trend for improvement in the GC and GD transplant recipients, but this trend did not reach statistical significance. The decrease in endurance with age and the lack of improvement or exacerbation of weakness supports the suggestion that it is not necessarily the germ cells influencing the strength as suggested in previous studies. No change from the transplants to the age-matched controls in total grip strength and endurance suggests that the musculoskeletal endurance may not be as influenced by reproductive status/hormones as other aspects of health and may exacerbate the decline in musculoskeletal conditions instead.

Another aspect of health related to musculoskeletal strength involves the grip strength of the forelimbs and the hindlimbs in mice. The classical dual grip strength test was used to assess specific strength rather than endurance. Grip strength in both the hind and forelimbs declined with age. The loss in hindlimb strength was exacerbated with the ovarian transplants. Unexpectedly, the GD transplants had the most significance in weakening the hindlimbs. The 600-day old GD transplant mice had similar hindlimb function as the 800-day control mice. Therefore, the reproductive status and physiological youth of the ovaries does not influence grip strength and endurance as well as we had hoped. However, we did not include sham surgery age-matched controls, which may have exposed a surgery-dependent loss of muscle strength due to the transplant procedure itself. This also suggests that HRT may have contradictory effects on the treatment of the quality and strength of muscle in humans. Further analysis of the actual hormone production and concentration between the groups should be analyzed to assess the influence of germ cell hormones on grip strength and endurance. Further musculoskeletal analysis on germ cell-depleted ovarian transplants may be necessary to further understand the role of ovarian somatic cells on strength and endurance. Considering this smaller sample size indicated potential but not statistical trends in endurance and decrease in strength, follow up studies may be necessary to gain a better grasp of this exacerbation in musculoskeletal conditions. One noteworthy aspect of the hindlimb grip strength test is the increase in strength in the 250-day control group compared to all other groups, including the youngest 150-day control group. This increase in hindlimb grip strength from the 150 to the 250 may be due the sheer size of the individual animal. This strain of mice continues to grow up until they are 500 days of age [9]. While their total weight may not fluctuate, their body changes and rate of growth are changing as they age. With body composition changes occurring and changing their size, rather than weight, may provide the 250-day old control group more strength in the hindlimbs considering they are developing into their peak weight.

Overall, young ovarian transplantation has a robust influence on health span. One limitation of this study is the lack of hormone concentration data available to assess the health span by the influence of germ cell dependent versus germ cell independent physiological pathways. Another limitation of this study is the modest number of animals available in each treatment group, particularly in the 700 to 800-day old group. Modest number of subjects is not an unusual situation in aging studies. Even with the moderate number of mice used in this study, significant differences and trends were still detected between group.

## 5. Conclusions

To summarize, sarcopenia is influenced by both aging and menopause. This loss in skeletal muscle can possibly be improved by the re-establishment of young ovarian influence in aged mice. These findings also reveal the perplexing concept of germ cell independent influence on sarcopenia and other aspects of health. Further investigation into the positive influences of young germ cell-depleted ovarian transplants can shed some light on the mechanisms of extending health. This further understanding has the potential to develop into an ovarian somatic cell related treatment for some post-menopausal and aging associated conditions. The hope is to eventually understand the communication and interaction in the ovarian somatic and germ cells that restores health in post-reproductive females.

## Figures and Tables

**Table 1:** Control and Experimental Groups. The different groups of mice are all separate groups with no repeated measures. The different groups are located across the top and their ages corresponding to the assays and euthanasia are listed below. The assays include Grip Strength, Cling-grip Endurance, and body composition using magnetic resonance imaging (MRI). The GC indicates the germ cell-containing transplant recipient group and the GD indicates the germ cell-depleted transplant recipient group.

Treatment Groups		Control				Treatment	
Assay	Age	Young (*n* = 19)	Adult (*n* = 10)	Old (*n* = 3)	Age-Match (*n* = 5)	GC (*n* = 9)	GD (*n* = 11)
Grip	At Assay (±10 days)	160	260	800	600	600	600
Cling-grip		160	240	800	600	600	600
MRI		160	240	700	500	500	500
	At Euthanasia (±10 days)	200	300	900	700	700	700
